# Course of COVID-19 infection in patients with congenital adrenal hyperplasia

**DOI:** 10.3389/fendo.2024.1348130

**Published:** 2024-02-09

**Authors:** Rida Javaid, Ninet Sinaii, Sarah Kollender, Jay Desai, Amy Moon, Deborah P. Merke

**Affiliations:** ^1^ Department of Pediatrics, National Institutes of Health Clinical Center, Bethesda, MD, United States; ^2^ Biostatistics and Epidemiology Service, National Institutes of Health Clinical Center, Bethesda, MD, United States; ^3^ The Eunice Kennedy Shriver National Institute of Child Health and Human Development, Bethesda, MD, United States

**Keywords:** COVID-19, congenital adrenal hyperplasia, CAH, adrenal insufficiency, glucocorticoid, adrenal, adrenal and infection, stress dose

## Abstract

**Context:**

Patients with primary adrenal insufficiency due to congenital adrenal hyperplasia (CAH) are at risk for adrenal crisis during infectious illnesses. Increased risk of infection including COVID-19 has been variably reported.

**Objective:**

To evaluate COVID-19 illness outcomes and stress dose practices in a large cohort of patients with CAH during the first two years of the pandemic and compare observations of COVID-19 infection in patients with CAH to the general USA population.

**Methods:**

Between March 2020 and November 2022, patients with CAH followed at the National Institutes of Health Clinical Center were queried about COVID-19 infection during their routine visits. Cases of COVID-19 were compared to controls. COVID-19 infection rates and symptoms were compared to general USA population data from the Centers for Disease Control and Prevention.

**Results:**

Of 168 patient visits, there were 54 (32%) cases of COVID-19 infection, and 15 (28%) were pediatric. Overall an association was found between acquiring COVID-19 and obesity (p=0.018), and adults acquiring COVID-19 were on lower doses of fludrocortisone (p=0.008). Fewer cases of COVID-19 infection were reported in those receiving hydrocortisone or modified-release hydrocortisone compared to longer acting glucocorticoids (p=0.0018). In our CAH population, the pattern of COVID-19 infection rates and COVID-related symptomatology were similar to those observed in the general USA population. Most patients with the presumed alpha variant reported anosmia and ageusia, while gastrointestinal symptoms were commonly reported during the delta and omicron waves. Stress dosing occurred in 30/54 cases, and 7 received parenteral hydrocortisone. Two hospitalizations occurred; one pediatric and one adult, both with co-morbidities. There were 5 emergency room visits and no reported deaths.

**Conclusion:**

Patients with CAH with close follow-up do not appear to be at increased risk of acquiring COVID-19 or to have a more severe course of COVID-19 compared to the general USA population. Obesity may increase risk of acquiring COVID-19 in patients with CAH, and overall infection risk may be lower in those receiving short-acting and circadian glucocorticoid replacement therapy. Established age-appropriate guidelines for stress dosing during infectious illnesses should be used for patients with CAH and COVID-19. COVID-19 specific guidelines are not indicated. Clinical Trial Registration: ClinicalTrials.gov, identifier NCT00250159.

## Introduction

Congenital adrenal hyperplasia (CAH) is the most common genetic cause of primary adrenal insufficiency (PAI) and the most common cause of PAI during childhood (1, 2). Treatment is glucocorticoid and mineralocorticoid replacement, however circulating levels of adrenal steroids are often insufficient for physiological requirements during infectious illnesses predisposing patients to adrenal crisis (3, 4). Although risk of adrenal crisis is mitigated by stress dosing with increasing daily glucocorticoid dose during times of physiological stressors (5), adrenal crisis remains a leading cause of death in populations with PAI (3). At the peak of the Coronavirus 19 (COVID-19) pandemic, there was little known about the impact of COVID-19 infection on patients with endocrinopathies, and it was recommended that patients with adrenal insufficiency stress dose during an acute COVID-19 infection (6–9). Since that time, variable data has emerged regarding the risks and outcomes of COVID-19 in patients with PAI.

Prior studies have proposed that patients with PAI are at increased risk of infections, which are a major trigger for life-threatening adrenal crises (10–12). A higher risk of infection [respiratory, genitourinary (GU) and gastrointestinal (GI)] was reported in patients with Addison’s disease and CAH, specifically those on maintenance and/or supraphysiological glucocorticoid dosing (12). Inefficient innate immune response to infections has been described in patients with PAI and glucocorticoid dependence, but this study was conducted in patients with PAI from autoimmune adrenalitis and/or bilateral adrenalectomy and not CAH (13).

COVID-19 is a highly contagious infection caused by severe acute respiratory syndrome coronavirus 2 (SARS-CoV-2) that has led to a variety of clinical sequelae worldwide. Since 2019, risk factors increasing predisposition to acquiring COVID-19 and a severe illness course have been identified, including obesity, cardiovascular disease, mental illness, and age > 60 years (14–17). A multinational survey among patients with chronic adrenal insufficiency showed an increased relative risk of hospitalization after acquiring COVID-19 infection. Risk factors included having CAH, age > 40 years, male gender, pulmonary disease, and higher maintenance doses of glucocorticoids (18).

Our study aimed to evaluate clinical outcomes and stress dose practices during illness with COVID-19 in patients with CAH enrolled in a longitudinal Natural History Study. We also assessed the trends of COVID-19 infection in our CAH population compared to the general USA population, and investigated associations between clinical characteristics and acquiring COVID-19 infection during the first years of the pandemic.

## Methods

### Study design and subjects

Between March 2020 and November 2022, adults and children with glucocorticoid-dependent types of CAH enrolled in a Natural History Study (ClinicalTrials.gov Identifier no. NCT00250159) at the National Institutes of Health Clinical Center were queried about COVID-19 illness during their routine visits. The glucocorticoid-dependent types of CAH included classic salt-wasting (SW) 21-hydroxylase deficiency, classic simple virilizing (SV) 21-hydroxylase deficiency, nonclassic 21-hydroxylase deficiency due to p30L mutation (NCp30L), and P450 side chain cleavage (P450_SCC_) deficiency.

Patients with CAH who reported acquiring COVID-19 were asked to complete a brief survey about symptomatology, course of illness, COVID-19 testing, COVID-19 vaccination status at the time of infection, and stress dose practices. Patient responses were collected in-person, during a remote telehealth visit or via telephone. During a COVID-19 infection, patients were instructed according to our standard “Sick Day Rules” to increase the daily glucocorticoid dose with four times per day dosing, double the daily dose in a setting of moderate febrile illness, triple the daily dose with severe illness, and/or administer hydrocortisone sodium succinate intramuscular injection if oral glucocorticoid dosing was not tolerated or ineffective (5). These “Sick Day Rules” are written illness-related glucocorticoid dosing instructions provided to patients at every clinic visit.

Subsequent chart review was conducted to collect patient comorbidities, laboratory results, and medication dosing at the visit prior to the reported time of COVID- 19. Blood was collected for hormonal evaluation in the morning before taking medications. Data were also recorded for patients who had a clinic visit during this time period but did not report COVID-19 infection. A comparison of disease characteristics was conducted between CAH patients with and without COVID-19. The pattern of COVID-19 in our CAH cohort was compared to the USA population based COVID-19 trends using data from the Centers for Disease Control and Prevention (CDC) (19).

The study was approved by the National Institutes of Health Institutional Review Board and all patients (adults) or parents (children) provided written informed consent. Children at least 8 years old provided written assent.

### Laboratory assays

Quantitation of 17-hydroxyprogesterone (17OHP), androstenedione and plasma renin activity (PRA) were done using liquid chromatography/tandem mass spectrometry (LC-MS) at the NIH Clinical Center Department of Laboratory Medicine (Bethesda, MD). Laboratory evaluation was sometimes performed locally due to pandemic restrictions. These samples were processed via Endocrine Sciences (LabCorp, Calabasas Hills, CA, USA) laboratory where 17OHP, androstenedione and plasma renin activity were analyzed using tandem LC-MS, (Triple Quadrupoles technique).

### Clinical data and definitions

The daily glucocorticoid dose was defined as dose equivalent to hydrocortisone as previously described (20, 21) and calculated based on body surface area. All glucocorticoid formulations were represented as hydrocortisone equivalent: 5 times the prednisone/prednisolone dose, 6 times the methylprednisolone dose and 80 times the dexamethasone dose. Some patients also received a modified-release hydrocortisone formulation (22). The analysis of various types of glucocorticoids excluded patients who were not taking glucocorticoids, although sensitivity analysis did not yield different conclusions.

In our cohort, pediatric was defined as younger than 18 years. Obesity in adults was defined as body mass index (BMI) ≥ 30 kg/m^2^ (21, 23). In children below 2 years of age, obesity was defined as BMI percentile at or above 85^th^%ile, and in children older than 2 years of age obesity was defined by BMI percentiles above the 95^th^%ile (24). Our case definition was based on each distinct visit as the unit of analysis and independent observation. Because COVID-19 infection can recur after recovery, this follows CDC guidelines for distinguishing a new case from an existing case when >90 days have passed since the last positive result (25). There were six CAH patients with repeat COVID-19 infections and a median of 396 days between infections.

Comorbidities such as diabetes mellitus, smoking status, hypertension, asthma and psychiatric illnesses were documented per patient report and chart review.

Androstenedione and plasma renin activity levels were considered elevated when they resulted above the reference range for age and sex. 17OHP levels were categorized as previously reported (21).

### Statistical analysis

Quantitative variables were reported as frequency (percentage) or median and interquartile range [IQR; 25^th^-75^th^ percentiles]. Categorical data were compared between groups (e.g., cases *vs* controls; vaccinated *vs* not) using Fisher’s exact tests; ordinal data were compared using tests for trend. For some results, odds ratios (OR) and 95% confidence intervals (CI) were reported. Continuous data were assessed for assumptions of normality, and compared between groups using Wilcoxon rank sum tests. Time series analyses described the epidemic curves. General USA population data were downloaded from the CDC website (19). Data analyses were performed using SAS v9.4 (SAS Institute, Inc, Cary, NC).

## Results

Of the 168 CAH visits during the study duration, there were 54 (32%) cases of COVID-19 infection, and the majority (72%) were adult visits (**Table 1**). Approximately half (48%) were female, and ages ranged from 8 months to 74 years. The type of CAH was similar between those who acquired COVID-19 infection and those who did not, and the majority of patients had salt-wasting 21-hydroxylase deficiency (**Table 1**). Among comorbidities, obesity was a risk factor for acquiring COVID-19 infection (33.3% *vs.* 16.7%, p=0.018; OR 2.5, 95% CI 1.18, 5.29). Other co-morbidities did not differ between the two groups.

**Table 1:** Clinical and Biochemical Characteristics of Patients with Congenital Adrenal Hyperplasia.

Characteristics	COVID (n=54)	No COVID (n=114)	*P*-value
**Female, n (%)**	23 (42.6)	58 (50.9)	0.327
**Age, years [median, IQR]**	26.6 [17.5-33.9]	22.1 [13.5-29.3]	0.067
Age, years [median, IQR] n (%)			
**Adult**	31.2 [25.2-39.8] 39 (72.2)	28.4 [24.7-34.2] 69 (60.5)	0.271
**Peds**	11.5 [8.4-17.2] 15 (27.8)	12.0 [7.3-16.8] 45 (39.5)	0.918
Phenotype, n (%) SW vs other phenotypes			0.103
SW SV SCC NC (P30L)	33 (61.1) 16 (29.6) 3 (5.6) 2 (3.7)	85 (74.6) 29 (25.4) 0 0	
**Obesity ^a^, n (%)**	18 (33.3)	19 (16.7)	0.018
**Asthma ^b^, n (%)**	2 (3.7)	4 (3.5)	1.000
**Diabetes ^b^, n (%)**	2 (3.7)	9 (7.9)	0.506
**Smoker ^c^, n (%)**	5 (9.3)	13 (11.4)	0.793
**Hypertension ^b^, n (%)**	4 (7.4)	5 (4.4)	0.471
**Depression/Anxiety ^b^, n (%)**	15 (27.8)	45 (39.5)	0.169
**GC dose (mg/m^2^/day), median [IQR] ^d^**	14 [10-17]	14 [11-18]	0.403
**Adults** **Peds**	15 [12-18] 12 [8-14]	15 [11-20] 13 [10-15]	0.602 0.137
Type of GC, n (%)			0.001
**Hydrocortisone** **Modified-release hydrocortisone** **Prednisone/prednisolone/methylprednisolone** **Dexamethasone** **Combination** **No glucocorticoids**	20 (37.0) 1 (1.9) 21 (38.9) 6 (11.1) 2 (3.7) 4 (7.4)	64 (56.1) 14 (12.3) 24 (21.1) 9 (7.9) 3 (2.6) 0 (0)	
**Fludrocortisone dose (mcg/day), median [IQR]**	100 [50-125]	100 [100-175]	0.009
**Adult** **Peds**	100 [75-150] 100 [50-100]	150 [100-200] 100 [75-125]	0.008 0.203
17-OHP ^e^, n (%)			0.843
**<100 ng/dL** **≥100-<1200 ng/dL** **≥1200-<5000 ng/dL** **≥5000 ng/dL**	7 (13.0) 18 (33.3) 15 (27.8) 14 (25.9)	13 (11.5) 39 (34.5) 30 (26.6) 31 (27.4)	
**Androstenedione elevated ^e,f^, n (%)**	20 (37.7)	30 (27.5)	0.364
**Plasma renin activity elevated ^e,f^, n (%)**	24 (48.0)	39 (35.8)	0.142

n = number of CAH cases.

^a^ Defined as body mass index ≥ 30 kg/m^2^ for adults, >95% in pediatric patients ≥2 years, >85^th^% in pediatric population < 2 years; ^b^ patient reported; ^c^ patient reported past and current smoking and/or vaping nicotine and/or marijuana products; ^d^ glucocorticoid dose equivalent to hydrocortisone in mg/body surface area/day;

^e^ Hormone levels collected closest to reported cases/clinic visits during study duration.

^f^ Above the normal range for age and sex.

GC, glucocorticoid; IQR, interquartile range; SW, salt-wasting; SV, simple virilizing; SCC= P450 side-chain cleavage enzyme deficiency; NC(p30L), nonclassic congenital adrenal hyperplasia due to p30L genotype.

Compared to patients receiving long-acting glucocorticoids, patients on hydrocortisone or a modified-release formulation of hydrocortisone (Chronocort^®^) had a lower rate of COVID-19 infection (42 *vs*. 68%, p=0.0018; OR 0.33, 95% CI 0.17, 0.66). All four patients who were nonadherent and reported not taking glucocorticoid therapy acquired COVID-19. Adults with COVID-19 infection were on lower doses of fludrocortisone (median 100 [IQR 75-150] *vs.* 150 [IQR 100-200], p=0.008). The degree of disease control, measured by biomarkers 17OHP, androstenedione and PRA, was not associated with acquiring COVID-19 infection (**Table 1**).

**Figure 1:**
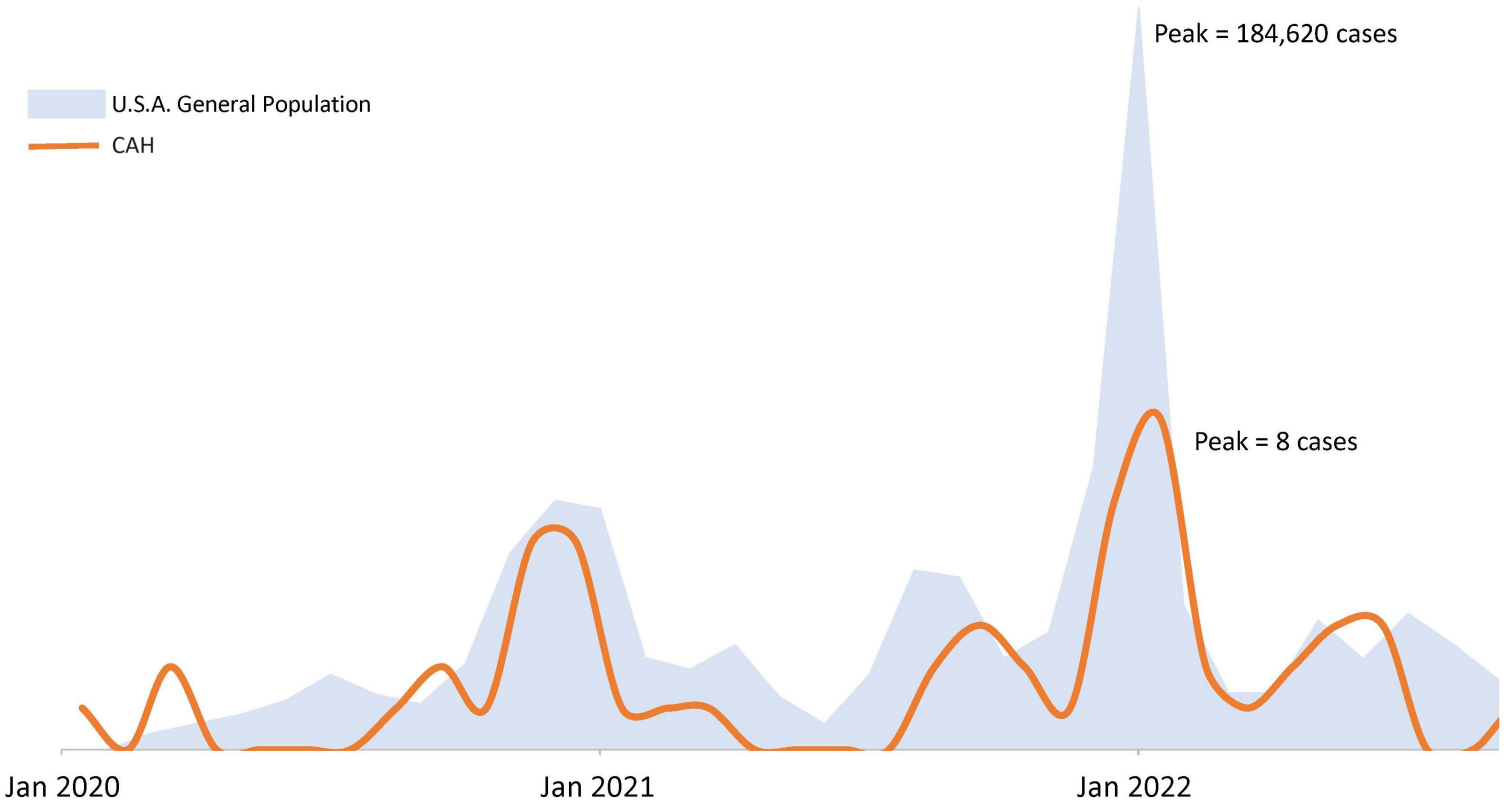
COVID-19 epidemic curves in a population of patients with congenital adrenal hyperplasia and the U.S.A. population. Figure not drawn to scale. USA population trends of COVID-19 infection based on case surveillance data from the Centers of Disease Control^20^.

**Figure 2:**
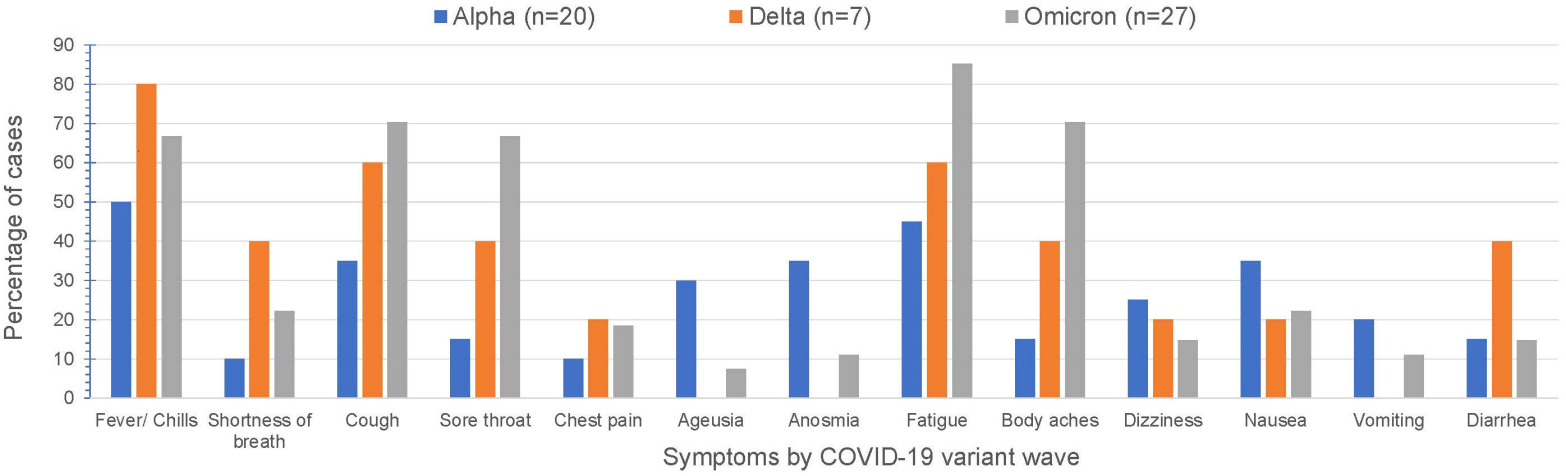
Symptomatology based on presumed circulating variants.

Over time, COVID-19 infection rates in our CAH population mirrored the USA population trends of COVID-19 infection (**Figure 1**). Reported symptoms were mild and similar to the general population based on presumed circulating variants. Cough, sore throat, fatigue and body aches were more prominent symptoms during both the delta and omicron surges (p=0.047, 0.008, 0.009, 0.0004 respectively). Cases during alpha circulation reported more anosmia and ageusia compared to cases with delta and omicron, while presumed delta and omicron cases were associated with more GI symptoms (**Figure 2**).

Six (11%) of 54 reported cases did not undergo COVID-19 testing to confirm diagnosis due to lack of available testing, but reported significant contact and COVID-like symptoms. Of the 48 (89%) cases that did get tested for COVID-19 infection, 45 (94%) were confirmed to be positive for the illness. Two of the 45 positive patients were asymptomatic. The remaining 3 cases were presumed positive based on symptomatology and contact.

The median duration of COVID-19 infection related symptoms was 5 days [IQR 3-7]. Patients took stress doses of glucocorticoid in 30 (56%) of 54 cases; and intramuscular Solu-Cortef was administered in 7 COVID-19 cases. Overall, the median duration of stress dosing was 1 day [IQR 0-3]. There were 5 emergency room visits. One pediatric and one adult patient were hospitalized during the study duration. The hospitalized pediatric patient was an 8 years old female with salt-wasting CAH, a seizure disorder, and developmental delay. Her symptoms were gastrointestinal comprising of emesis and diarrhea, and she received intramuscular injection of Solu-Cortef. Duration of hospitalization was 2 days. The hospitalized adult patient was a 63 years old female with simple virilizing CAH and a history of hypertension, chronic kidney disease, obesity and diabetes mellitus. She presented to an emergency room with dyspnea, hypoxia, coughing, and fevers, and was hospitalized for 8 days. She did not require intubation or intensive care, but did receive supplemental oxygen. There were no reported patient deaths.

About half (25 of the 54, 46%) of reported COVID-19 cases were in vaccinated patients at the time of the infection. No differences were observed in reported symptomatology or stress dosing practices between those vaccinated and those not. However, four (80%) out of five visiting the ER were unvaccinated at the time of illness (p=0.019). Neither of the two hospitalized patients were vaccinated.

## Discussion

In our longitudinal Natural History study, we found that adult and pediatric patients with primary adrenal insufficiency due to CAH who acquired COVID-19 infection during the first two years of the pandemic had similar symptomatology and course of illness as the general USA population. Patients with CAH who acquired COVID-19 infection had a higher prevalence of obesity, a known risk factor for severe illness with COVID-19, and adults were on lower doses of fludrocortisone compared to CAH patients who did not acquire COVID-19. Fewer cases of COVID-19 infection were observed in patients receiving hydrocortisone or modified-release hydrocortisone for maintenance glucocorticoid therapy, emphasizing the potential compromising effects of long-acting glucocorticoids in the treatment of adrenal insufficiency. Standard stress dosing practices based on degree of symptomatology were utilized and were sufficient in preventing adrenal crisis.

A higher incidence of infections in patients with adrenal insufficiency and CAH compared to controls has been reported. In a population-based retrospective study of primary care data from 1995 to 2018, CAH and Addison’s disease patients were found to have increased incidence of respiratory, gastrointestinal and genitourinary infections, and those not receiving glucocorticoid did not have an increased risk of infection (12). A cross-sectional study of 42 patients with PAI due to autoimmune adrenalitis or bilateral adrenalectomy found that innate immune function, specifically natural killer cell cytotoxicity, was compromised in patients with PAI compared to age- and sex-matched controls suggesting vulnerability to viral infections (13). Our findings do not support this. In our CAH population, the patterns of COVID-19 infection rates and COVID-related symptomatology were similar to the general USA population. Our patients with CAH did not appear to be at increased risk of acquiring COVID-19 or to have a more severe course of COVID-19 compared to the general population.

Our findings support the concept that the type of glucocorticoid and method of delivery may impact risk of viral infection in patients with PAI due to CAH. Similarly, in a cross-sectional single center study of patients with PAI in the UK, those who reported COVID-19 were less likely to be on immediate release glucocorticoid replacement or hydrocortisone therapy (26). In our study, the majority of our patients were receiving standard multi-dose glucocorticoid, with a variety of regimens. Overall, we observed fewer COVID-19 cases in our population of CAH patients receiving hydrocortisone and circadian hydrocortisone replacement with modified-release hydrocortisone suggesting that short-acting glucocorticoid replacement therapy and near-physiological circadian replacement may reduce risk of infection in CAH patients. In a single-blind randomized controlled trial, patients with primary adrenal insufficiency receiving once-daily modified-release glucocorticoid (Plenadren^®^) had more normal monocyte and natural killer cell immune profiles and reduced infections compared to a standard multi-dose glucocorticoid regimen, suggesting that circadian glucocorticoid replacement may reduce rate of infection in PAI patients (27, 28). Interestingly, our patients who were non-compliant and no longer taking glucocorticoid or mineralocorticoid replacement therapy all reported COVID-19 infection.

We found increased risk of COVID-19 in adults with CAH receiving lower mineralocorticoid replacement dosage, suggesting a possible role of salt-wasting in susceptibility to infection. The tendency of practitioners to lower fludrocortisone dose in adults to avoid hypertension might increase the risk of salt wasting during illnesses. Mineralocorticoid replacement is recommended in all patients with classic CAH, and volume depletion and salt loss from insufficient mineralocorticoid replacement might influence risk of adrenal crisis (1). Similarly, patients with PAI and COVID-19 have been found to be less commonly treated with fludrocortisone (26). The relationship between mineralocorticoid dosage and risk of infectious illness requires further study.

COVID-19 outcomes in patients with PAI including CAH have been studied (18, 26, 29) A multinational survey between September and December 2020 among patients with chronic adrenal insufficiency showed an increased relative risk of hospitalization after acquiring COVID-19 infection. Risk factors included diagnosis of congenital adrenal hyperplasia, age > 40 y, male gender, pulmonary disease, and higher maintenance doses of glucocorticoids (18). Another multicenter European study aimed to determine the outcomes of COVID-19 infection in patients with adrenal disorders, including primary and secondary adrenal insufficiency as well as Cushing’s syndrome, between January 2020 and December 2021. Among the 64 cases identified, 45 had primary adrenal insufficiency and 19 had congenital adrenal hyperplasia, 92% achieved full remission, 6% had persisting sequalae and 2% had lethal outcome. Patients with CAH were found to have the highest rate of COVID-19 remission (29) consistent with our findings of good clinical outcomes.

Co-existing conditions play an important role in COVID-19 outcomes. Epidemiological data derived from USA demonstrated that COVID-19 related hospitalization was six times higher in individuals with pre-existing conditions including; cardiovascular illness, obesity, diabetes mellitus, smoking, and cancer, and such individuals were at high risk of COVID-related morbidity and mortality (14, 15). Males more than females were found to have a more severe course of illness (14), and obesity has been found to be associated with increased severity and higher mortality among COVID-19 patients (30). In general, our patients with CAH and COVID-19 fared well and were not hospitalized. Our one adult (63 y female) who was hospitalized had pre-existing diabetes, obesity, cardiovascular disease and chronic kidney disease, and the one child hospitalized had neurological delay, a pediatric condition associated with increased risk of a severe COVID-19 course (17). We found obesity to be a risk factor for acquiring COVID-19 among patients with CAH. The possible association between healthy lifestyle practices during the pandemic and choice of CAH therapy was not assessed, but may have played a role. Unlike population-based studies, we did not find a sex difference in COVID-19 incidence or course severity.

Patients with CAH due to 21-hydroxylase deficiency are more susceptible to developing risk factors for cardiovascular disease and metabolic syndrome, both during childhood and adulthood (21, 31). In a recent retrospective study of cardiovascular and metabolic risk in a cohort of 254 patients with CAH, adults who had a history of cardiovascular disease were on a regimen of higher glucocorticoid dose than those without CVD (31). Overall, patients with CAH are susceptible to cardiovascular disease risk factors such as obesity, insulin resistance, hypertension and diabetes, and dexamethasone is associated with more adverse effects, especially obesity, compared to those treated with prednisone or hydrocortisone (32). Thus, in addition to potential adverse effects of long-acting glucocorticoid on the immune system, longer acting glucocorticoid also increases risk of metabolic comorbidities that per se are risk factors for COVID infection and worse outcome.

In patients with PAI, adrenal crisis is most often precipitated by infectious illnesses (1, 3, 5, 33). Proper education on timely administration of immediate release glucocorticoid therapy can help prevent acute events from COVID-19 infections in patients with PAI (6, 7, 34–36). In our cohort, about 55% of reported cases initiated stress dosing during COVID-19 infection, only 13% of cases received parenteral glucocorticoid administration, and no COVID-19 related deaths were reported. In general, our patients did dose adjustments according to our “Sick Day Rules” instructions.

In our study, the majority of reported COVID-19 cases (54%) were not vaccinated at the time of infection. Our data collection started at least one year before the COVID-19 vaccination was available which could explain this finding. There may also have been some vaccine hesitancy, although our study did not address this.

Our study was conducted in a large CAH cohort in the USA being followed longitudinally providing an opportunity to review stress dosing practices, COVID-19 outcomes, and analyze how various CAH characteristics and other comorbidities impacted the course of COVID-19 in patients with adrenal insufficiency due to classic CAH. The trend of COVID-19 infection in our cohort was similar to that in the general US population. We did not find any aspect of COVID-19 symptoms to be uniquely related to CAH.

Our study has several limitations and could have been strengthened further by confirmation of COVID-19 via PCR in each reported case. Symptoms were patient-reported and cases were not clinically evaluated at the time of infection in person given the pandemic restrictions. Survey questions were asked via telephone. Only patients who had a clinic visit during the study time period participated. Some of the laboratory values were obtained more than 1 month prior to the reported case of infection. Laboratory data obtained closer to the reported COVID-19 infection would have been more informative regarding endocrine status. Inclusion of nonclassic CAH patients could have helped ascertain whether daily glucocorticoid therapy increases predisposition for COVID-19 infection. The status of COVID-19 vaccination in the control group could have helped stratify the risk of acquiring COVID-19 in our cohort after COVID-19 vaccines were available. Variable availability of testing may have underestimated our reported COVID-19 infection rates. In addition, socioeconomic status was not evaluated.

Our findings highlight the importance of the daily maintenance therapy regimen in contributing to risk of infectious illness in patients with CAH. Short-acting glucocorticoid therapy and circadian replacement might reduce risk. Studies are needed to better comprehend the role of physiological circadian dosing of glucocorticoid replacement and mineralocorticoid dosing in the immunity of patients with CAH. Obesity, a common co-morbidity in CAH, may increase risk of acquiring COVID-19. Patients with CAH should be educated regarding standard age-specific stress dosing practices based on degree of symptomatology for COVID-19 infection. COVID-19 specific guidelines are not indicated. Patients with CAH remain at risk of adrenal crisis as with any infectious illness. Preventative measures, such as immunization and self-protective behavior, are recommended in addition to glucocorticoid stress dosing if indicated according to standard sick day rules.

## Data availability statement

The original contributions presented in the study are included in the article/supplementary material. Further inquiries can be directed to the corresponding author.

## Ethics statement

The studies involving humans were approved by the National Institutes of Health Institutional Review Board. The studies were conducted in accordance with the local legislation and institutional requirements. Written informed consent for participation in this study was provided by the participants, or for children by the participants' legal guardian/next of kin.

## Author contributions

RJ: Conceptualization, Data curation, Investigation, Methodology, Project administration, Writing – original draft. NS: Formal analysis, Methodology, Software, Supervision, Validation, Visualization, Writing – review & editing. SK: Data curation, Methodology, Writing – review & editing. JD: Methodology, Writing – review & editing. AM: Writing – review & editing. DM: Conceptualization, Funding acquisition, Project administration, Resources, Supervision, Validation, Visualization, Writing – review & editing.
